# Applications of Bionano Sensor for Extracellular Vesicles Analysis

**DOI:** 10.3390/ma13173677

**Published:** 2020-08-21

**Authors:** Jin-Ha Choi, Jin-Ho Lee, Jeong-Woo Choi

**Affiliations:** 1 Department of Chemical and Biomolecular Engineering, Sogang University, Seoul 04107, Korea; jinhachoi@sogang.ac.kr; 2 School of Biomedical Convergence Engineering, Pusan National University, Yangsan 50612, Korea

**Keywords:** biosensors, extracellular vesicle, nanoparticle, nanotechnology, optical sensor, electrochemical sensor

## Abstract

Recently, extracellular vesicles (EVs) and their contents have been revealed to play crucial roles in the intrinsic intercellular communications and have received extensive attention as next-generation biomarkers for diagnosis of diseases such as cancers. However, due to the structural nature of the EVs, the precise isolation and characterization are extremely challenging. To this end, tremendous efforts have been made to develop bionano sensors for the precise and sensitive characterization of EVs from a complex biologic fluid. In this review, we will provide a detailed discussion of recently developed bionano sensors in which EVs analysis applications were achieved, typically in optical and electrochemical methods. We believe that the topics discussed in this review will be useful to provide a concise guideline in the development of bionano sensors for EVs monitoring in the future. The development of a novel strategy to monitor various bio/chemical materials from EVs will provide promising information to understand cellular activities in a more precise manner and accelerates research on both cancer and cell-based therapy.

## 1. Introduction

Recently, extracellular vesicles (EVs) (50–200 nm in diameter) have emerged as a new biomarker to diagnose disease progression and monitor treatment efficacy because of their vital role in cell–cell communication and molecular exchange [1,2,3]. These cell-derived membrane-enclosed vesicles are physically stable, abundant in biological fluids (e.g., >10^10^ EVs/mL of blood) and carry cell-specific cargos that are characteristic of their cells of origin, including proteins and nucleic acids (e.g., micro RNA (miRNA), messenger RNA (mRNA) and DNA) [4,5,6,7]. Typically, extracellular vesicles are enriched with a transport or fusion protein (e.g., caveolin-1), tetraspanins (e.g., CD63, CD9, CD81) and heat-shock proteins (Hsp 60, Hsp 70 and Hsp 90) due to the endosomal formation of vesicles [8]. Most of the nucleic acids (approximately 76% of the total oligonucleotides) inside EVs are non-coding RNAs (i.e., miRNAs), which are involved in many different roles and functions of cells as post-transcriptional gene regulators [9,10]. Furthermore, because EVs are actively secreted in large quantities by cells, they are promising biomarker candidates for the early diagnosis of diseases and sub-sequential monitoring of the therapeutic response from liquid biopsies in a minimally invasive manner [11].

Despite such clinical potential, the reliable isolation and analyses of EVs from complex biologic fluids are enormously challenging because of the small size and low density of these biomolecules, thereby necessitating an extensive sample preparation technique before measurements. Although ultracentrifugation remains the gold standard for isolation of extracellular vesicles, it still requires expensive instruments, tedious steps and may introduce some impurities [5,12]. Meanwhile, conventional analytical methods, including western blotting, enzyme-linked immunosorbent assay (ELISA) and flow cytometry, have also been facing technical hurdles owing to the limited sample volume, low sensitivity, and/or requirement of specialized high-end equipment [13,14,15,16,17]. For example, the quantification of EVs by ELISA has a limit of approximately 10^4^ in 100-μL of the sample [18]. To improve the efficiency and sensitivity of EV isolation from a complex biologic fluid, various nanotechnology-based biosensors have been developed [19,20,21].

This review describes some of the current state-of-the-art developments in nanotechnology-based biosensor platforms for analyzing exosomal proteins and RNAs. These approaches are classified into four broad categories: surface plasmon resonance (SPR), colorimetric/fluorescence, Raman and electrochemical. The topics discussed in this review provide some insight into and guidelines for developing a nanotechnology-based EV characterization platform to promote the translation of EVs into clinical applications.

## 2. Surface Plasmon Resonance-Based Analysis Methods for EV Detection

The SPR-based analytical method is an efficient platform for detecting and characterizing molecular interactions between biologic molecules [22,23,24,25,26]. This method monitors local refractive index changes generated by the resonant oscillation of stimulated electrons near the sensing surface (i.e., nanoscale metal materials) upon the binding of a ligand to a target molecule [27,28,29,30,31]. Owing to this simple and distinctive phenomenon, SPR-based biosensors have gained extensive attention to develop label-free and real-time biosensors with minimal sample preparation procedures [32,33,34,35,36]. Furthermore, as the dimension of EVs perfectly matches the sensing depth of SPR (200 nm), SPR-based biosensors are effective for quantifying EVs from complex biologic fluids [37,38,39,40]. In this section, recent studies related to SPR-based exosomal detection platforms are briefly introduced.

**Figure 1:**
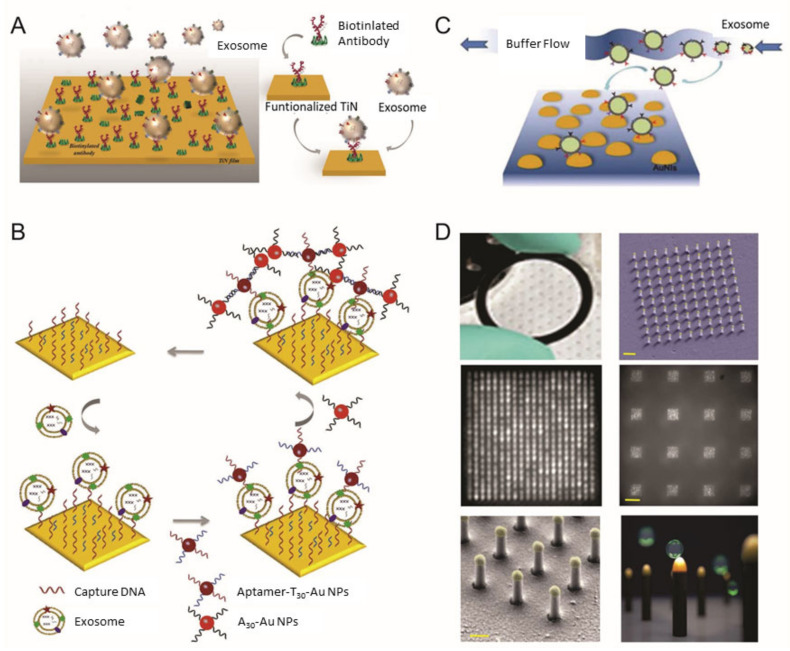
Surface plasmon resonance (SPR) biosensor for extracellular vesicles (EV) detection. (**A**) Schematic illustration of TiN nano-thick film functionalized by the anti-CD63 antibody for the detection of EVs; (**B**) dual Au Np-assisted signal amplification for the determination of EVs; (**C**) biophysical interaction of EV with self-assembled Au NIs without functionalization; (**D**) Au nanoplasmonic array functionalized with the anti-CD63 antibody for LSPR based digitalized detection of the EV. (reproduced with permission from [41] published by WILEY-VCH 2019, reproduced with permission from [42] published by Elsevier 2019, reproduced with permission from [43] published by Elsevier 2017 and reproduced with permission from [44] published by Public Library of Science 2018).

Qiu et al. utilized a nano-thick titanium nitride (TiN) film as an alternative plasmonic material in the SPR biosensing system for sensitive detection of EVs derived from malignant glioma cells (U251) (Figure 1A) [41]. Based on the high affinity between the TiN film surface and biotin, the TiN film was functionalized by biotinylated anti-epidermal growth factor receptor (EGFR) variant-III (EGFR vIII) antibodies or anti-CD63 to capture U251-derived EVs. Both an in vitro cell culture condition and serum were tested to validate the performance of the biosensor in a real sample condition (i.e., complex biologic fluid). The developed biosensor had a limit of detection (LOD) of 4.29 × 10^−3^-µg/mL for a general EV marker (CD63) and 2.75 × 10^−3^-µg/mL for the glioma cell-specific marker, EGFR vIII. Compared with well-known plasmonic metal materials, including gold (Au), TiN even demonstrated better sensitivity, while having a tunable plasmonic property in the visible to near-infrared spectrum range as well.

In addition to detecting classic antigen–antibody interactions, the SPR-based approaches have proved useful for the sensitive and selective determination of EVs based on aptamer recognition. For example, Wang et al. demonstrated how a sensitive aptasensor could be used for EV detection by SPR with controlled two-step hybridization of Au nanoparticle (NP)-assisted signal amplification (Figure 1B) [42]. Briefly, aptamer CD63 that targets the general EV surface marker CD63 was functionalized on surfaces of Au film to capture EVs. The addition of Au NPs modified with two different aptamers (CD63 and T30, where T30 is 5′-TTT (×10)-3′) led the first signal amplification by forming a sandwich structure “CD63-aptamer-modified Au film/EV/CD63 aptamer and T30-aptamer-modified Au NPs”. A second signal amplification was obtained by employing aptamer A30 (5′-AAA (×10)-3′)-functionalized Au NPs to capture the T30 sequence by the complementary base pairing procedure based on the A–T interaction. The consequent electronic coupling between (1) the Au film and Au NPs and (2) the Au NPs and Au NPs changed the local refractive index, separately, enabling dual-signal amplification. By utilizing this dual Au NP-assisted signal amplification, the authors achieved high sensitivity and low LOD (5 × 10^3^ EVs/mL), corresponding to a 10^4^-fold improvement compared with ELISA. Moreover, the developed SPR-based sensor system could detect the EVs in complex biologic fluids (30% fetal bovine serum) and differentiate the EVs secreted by two different cell lines (MCF-10A normal breast cells and MCF-7 breast cancer cells).

Alternatively, Im et al. reported an on-chip nanoplasmonic EV sensor (nPLEX) for high-throughput EV protein analysis based on the localized surface plasmon resonance (LSPR) mechanism [45]. The chip comprises an Au layer with periodic nanohole arrays functionalized with different antibodies for binding specific EVs and profiling of EV surface proteins and proteins present in EV lysates (71 protein biomarkers in ovarian cancer and benign cells were analyzed). In detail, the Au nanoholes were designed with a probing depth of less than 200-nm to match the size of the EV (<100 nm) to improve sensitivity. With target-specific binding of EV, the spectral intensity or shifts changes proportional to the target marker protein levels were successfully obtained, and the LOD of the nPLEX chip was estimated to be 3000 EVs/mL (670 aM). In addition, when samples from cancer patients were analyzed, ovarian cancer correlated with the expression of CD24 and epithelial cell adhesion molecule (EpCAM).

Thakur et al. used a self-assembled array of Au nanoislands to develop a sensitive and low-cost LSPR-based biosensors for detecting EVs isolated from A-549 cells, SH-SY5Y cells, blood serum and urine from a lung cancer mouse model (Figure 1C) [3]. The active structure of the Au nanoislands array was constructed by deposition and annealing procedures and utilized for the detection of EVs without any surface functionalization. The LOD of this sensor was 0.194 µg/mL. Similarly, Bathini et al. demonstrated an Au nanoislands-assisted EV detection system functionalized with a peptide called Venceremin (Vn96) [46]. Vn96 is a synthetic peptide with a strong affinity for heat-shock proteins on the surface of EVs. With a simple physical model, the optimum streptavidin–biotin–polyethylene glycol–Vn96 complex formation condition was achieved and experimentally optimized in agreement with the physical model. It was found that the surface of each Au nanoislands could accommodate nine EVs, resulting in 27 EVs/μm^2^.

In another example, Raghu et al. fabricated Au nanoplasmonic pillar arrays by combining nano- and microfabrication techniques for single EV detection (Figure 1D) [44]. By limiting the size of the individual elliptically shaped nanoplasmonic pillar similar to the size of an EV (approximately 100 nm), in situ EV binding events (secreted by MCF7 breast adenocarcinoma cells) were observed, even at sub-femtomolar concentrations. Recent studies of SPR-and LSPR-based analytical methods for detecting EVs are compared in Table 1.

## 3. Colorimetric/Fluorescence-Based Analysis Methods for EV Detection

**Figure 2:**
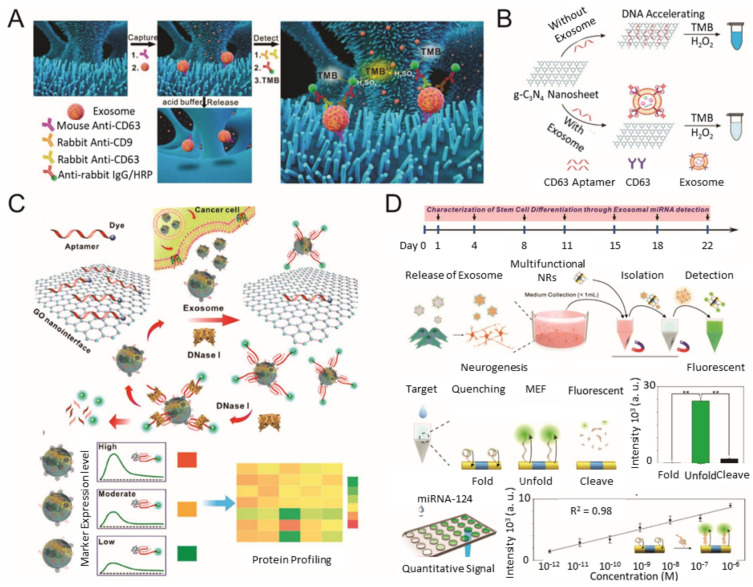
Colorimetric/fluorescence biosensor for EV detection. (**A**) Schematic diagram of the ZnO-nanowires-coated three-dimensional (3D) scaffold chip for EV detection; (**B**) utilization of peroxidase-like activity of graphitic carbon nitride nanosheets (g-C3N4 NSs); (**C**) fluorescence resonance energy transfer (FRET) biosensing system based on aptamer and graphene oxide (GO); (**D**) multifunctional nanorods (NRs) based exosomal miRNA signal amplification based on metal-enhanced fluorescence effect. (reproduced with permission from [52] published by Elsevier 2018, reproduced with permission from [54] published by American Chemical Society 2017, reproduced with permission from [55] published by American Chemical Society 2018 and reproduced with permission from [63] published by American Chemical Society 2019).

During the past few years, various types of nanomaterials with unique optical properties have been extensively applied to develop sensitive biosensors for EV analysis [21,47]. Among the optical techniques, a promising nanotechnology-based colorimetric biosensor has been established to detect exosomal biomarkers with the naked eye based on the extinction coefficient [48,49,50]. In general, nanotechnology-based colorimetric biosensors can be characterized into two groups based on their different properties of nanomaterials: catalytic properties (i.e., nanozymes) and inherent optical properties [51]. As an example of the catalytic property, Chen et al. improved the sensitivity of the traditional ELISA by fabricating a three-dimensional (3D) zinc oxide (ZnO) nanowires-coated scaffold chip device (Figure 2A) [52]. The presence of a hierarchical nanointerface, which was obtained by a 3D polydimethylsiloxane scaffold and free-standing ZnO nanowires, provides high capture efficiency of EVs as a result of the large surface area, as well as a size exclusion-like effect. After the isolation, a colorimetric assay based on horseradish peroxidase (HRP)-labeled antibody and 3,3′,5,5′-tetramethylbenzidine was performed for the quantitative analysis of EVs. Based on the improved sensitivity compared with a commercial ELISA, down to 2.2 × 10^4^ EVs/μL was detected with a linear range of 2.2 × 10^5^–2.4 × 10^7^ EVs/μL. Likewise, Zhang et al. integrated a microfluidic chip with self-assembled 3D herringbone nanopatterns to promote microscale mass transfer by increasing the surface area for efficient EV isolation [53]. Combined with a colorimetric method, the LOD achieved was down to 10 EVs/mL. Moreover, the quantitative detection of circulating exosomal CD24, EpCAM and folate receptor–alpha protein for diagnosing ovarian cancer was demonstrated using only 2 μL of plasma.

As an alternative approach to improve sensitivity for EV detection, Wang et al. utilized the inherent peroxidase-like activity of graphitic carbon nitride (g-C_3_N_4_) nanosheets for a colorimetric assay (Figure 2B) [54]. In addition to the peroxidase-like activity of g-C_3_N_4_ nanosheets, the adsorption of single-stranded DNA (ssDNA) was also found to improve the catalytic activity. The maximum reaction rate of the hydrogen peroxide-mediated 3,3′,5,5′-tetramethylbenzidine reaction in the presence of ssDNA–g-C_3_N_4_ nanosheets was at least four-fold faster than pure g-C_3_N_4_ nanosheets. Considering this advantage, aptamer CD63 was introduced to specifically target EVs and generate variable catalytic ability (decrement) based on the varying concentrations (increment) of EVs. The EVs concentration was quantified by the color change of the product, and the LOD was calculated to be down to 13.52 × 10^5^ EVs/mL. A similar signal trend was also observed from EVs collected from the sera samples. The developed system could also be used to characterize the differential expression of exosomal CD63 between the breast cancer cell line (MCF-7) and a control cell line (MCF-10A). Xia et al. quantified EVs based on aptamer-CD63-functionalized single-walled carbon nanotubes (s-SWCNTs) [56]. As the functionalization with ssDNA exhibited enhanced peroxidase activity of s-SWCNTs, the addition of EVs resulted in a decrement of catalytic ability and a color change as well. The LOD was 5.2 × 10^5^ EVs/μL, which was 10-fold lower than the commercial ELISA. Similar to this, Chen et al. detected prostate cancer EVs by integrating EpCAM aptamer on iron oxide (Fe_3_O_4_) NPs, which also possess peroxidase activity [57]. Prior to the detection of the EV, an anion exchange-based isolation (with magnetic beads) was performed to collect EVs from plasma and cell culture medium. Through the suggested method, the EVs were isolated with >90% efficiency and presented fewer protein impurities compared with ultracentrifugation. Applying the EpCAM aptamer–Fe_3_O_4_ NPs for colorimetric detection of prostate cancer EVs revealed a linear range from 0.4 × 10^8^ to 6.0 × 10^8^ EVs/mL and an estimated LOD of 3.58 × 10^6^ EVs/mL.

Instead of using the catalytic property of nanomaterials, Jiang et al. reported a multiplexed EV sensing system based on the inherent optical properties of Au NPs [58]. The sensor system was composed of Au NPs complexed with a panel of aptamers. The surface capped with aptamers protected the Au NPs against aggregation/agglomeration in a high-salt solution and helped to maintain the colloidal stability. When EVs are presented, the stronger binding between the aptamer and targeted surface protein EV dominates over the non-specific binding between aptamers and the Au NPs. Subsequent electrostatic aggregation/agglomeration of Au NPs occurs, and the solution changes from red to blue because of the detachment of the aptamer from the surface of Au NPs. By employing this simple approach, the differential expression of surface proteins (CD63, EpCAM, platelet-derived growth factor, prostate-specific membrane antigen, protein tyrosine kinase-7) was investigated in EVs derived from human cervical carcinoma (HeLa), human acute lymphoblastic leukemia (Ramos), human prostate cancer (PC3) and human acute lymphoblastic leukemia (CEM) cells. Liu et al. detected EV released from nasopharyngeal carcinoma cells by applying a simple approach [59]. This system employed two proximity-ligation-assay probes to generate a unique surrogate DNA signal for EV detection. The DNA signal was multiply amplified by transcription-mediated amplification united with recombinase polymerase amplification and quantitatively detected by colorimetric assay using Au NPs. Through this multiple amplification, plasma Epstein–Barr virus latent membrane protein 1-positive (accuracy: 0.956) and EGFR-positive (accuracy: 0.906) EV levels in patients were investigated. This colorimetric sensor showed a LOD of 10^2^ EVs/mL and a wide dynamic range of 10^2^–10^8^ EVs/mL.

As an alternative approach, a nanotechnology-based fluorescence biosensor has been considered as a notable and popular detection technique with high sensitivity and selectivity [60]. For example, various nanomaterials, including two-dimensional nanomaterials, metal oxide NPs, up-conversion nanoparticles (UCNPs) and metallic NPs, have been employed in optical biosensors based on their distinctive optical properties [21,60]. Jin et al. constructed a fluorescence resonance energy transfer-mediated biosensing system based on an aptamer and graphene oxide (GO) to quantify prostate cancer EVs (Figure 2C) [55]. Owing to the complete atomic-thick layers and abundant terminal groups, GO exhibited a high affinity for the target-responsive fluorescent aptamer and intrinsic fluorescence quenching ability to form a quenched aptamer–GO nanoprobe. When the EVs are presented, the aptamer preferentially binds with EVs and restores the fluorescent signal via detachment from GO. In addition, the exposed aptamer sequence can be digested by deoxyribonuclease-I, so that the targeted surface protein of the EV can be recycled to amplify the fluorescence signal. As a result, the LOD achieved was 1.6 × 10^5^ EVs/mL. Furthermore, by modulating seven different aptamers (CD63, EpCAM, prostate-specific membrane antigen, protein tyrosine kinase-7, carcinoembryonic antigen, platelet-derived growth factor), the expression level on EVs derived from serum samples of prostate cancer patients and healthy individuals. Taking advantage of the similar absorption property of GO, Zhang et al. constructed a biosensing platform based on the Cy3-labeled aptamer-CD63 and Ti_3_C_2_ MXenes complex for EVs detection [61]. The selective adsorption of Cy3-labeled aptamer-CD63 on Ti_3_C_2_ MXenes via hydrogen bond and metal chelate interaction results in a fluorescence quenching of the labeled Cy3 dye. When EVs are presented, the fluorescent Cy3 label is recovered because of the relatively stronger affinity between the aptamer and EV. The linear range was estimated to be 10^4^–10^9^ EVs/mL, and the LOD was 10^2^ EVs/mL. Chen et al. developed a simple paper-supported biosensor based on luminescence resonance energy transfer from UCNPs to Au nanorods for EV detection [62]. Here, two aptamer fragments were designed to bind with the CD63 protein on the surface of EVs and recruit Au nanorods near the UCNPs, which initiates the luminescence quenching of the green fluorescence of UCNPs. As a result, the quenched fluorescent signal linearly correlated to the concentration range of 1.0 × 10^4^–1.0 × 10^8^ EVs/μL and reached a LOD of down to 1.1 × 10^3^ EVs/μL.

Lee et al. developed a selective and sensitive system to detect exosomal miRNA (Figure 2D) by using the quenching and recovery of fluorescence mechanism, as well as the metal-enhanced fluorescence of a multifunctional single nanorod structure with magnetic and plasmonic components [63]. Each component was specifically modified to selectively isolate targeted EVs through magnetic immunoseparation and sensitively and selectively identify targeted exosomal miRNA using a fluorophore-labeled hairpin single-stranded molecular beacon. The fluorescence signal from the miRNA−magnetic bead complex could be enhanced through metal-enhanced fluorescence effects at the Au component area of the multifunctional nanorod. Interestingly, this system was used to characterize the neurogenesis of stem cells, as well as a heterogeneous population of neural cells in an ex vivo rodent model, which indicates the importance of EVs in applications other than disease monitoring. Similarly, He et al. presented a copper-mediated fluorescent signal amplification strategy for EV detection [64]. Cholesterol-modified magnetic beads captured EVs via hydrophobic interactions between cholesterol moieties and lipid membranes. Subsequently, sandwich complexes were formed by aptamer-CD63-modified copper oxide (CuO) NPs. The consequential sandwich complexes are liquified by acidolysis to transform CuO NPs into copper (II) ions (Cu^2+^) and Cu^2+^ can be reduced to form fluorescent Cu NPs in the presence of sodium ascorbate and polythymine. As the fluorescence of Cu NPs is directly proportional to the concentration of Cu^2+^ and EVs, quantitative analysis was achieved in the range of 7.5 × 10^4^–1.5 × 10^7^ EVs/μL and LOD of 4.8 × 10^4^ EVs/μL in complex biologic fluids. Recent studies of colorimetry- and fluorescence-based analytical methods for EV detection are compared in Table 2.

## 4. Electrochemical-Based Analysis Methods for EV Detection

Electrochemical biosensors hold prodigious potential in the biomedical field because of their high specificity, sensitivity, label-free and cost-effectiveness for detecting several biomarkers of diseases [66,67,68,69]. These devices contain biologic recognition elements with electrochemical signaling molecules such as enzymes [70], antibodies with electro-active molecules [71] and nucleic acids with electro-active molecules [72] that selectively responds with the target biomolecules and produces an electrical signal that is related to the concentration of the target analyte. In an electrochemical biosensor, the transducer translates biologic events, such as an immune reaction or a nucleic acid hybridization, into an electrical signal. There are representative analytical methods for the detection of biologic events, which are amperometry, potentiometry, voltammetry and impedance spectroscopy [73,74]. Among them, amperometry measures the change of current with constant potential, whereas potentiometric analysis converts biologic events to the equilibrium potential difference. In addition, voltammetry was shown the current change with varying voltages. These strategies utilized the reduction and oxidation of the specific redox materials, whose redox properties could be changed due to the biologic reaction. Owing to this phenomenon, electrochemical biosensors operate extensively in disease diagnostics for the detection of suitable markers, including EVs, proteins and nucleic acids. In addition, electro-active nanomaterials have been applied to electrochemical biosensors for improving the performance, including sensitivity and specificity. This section discusses current studies on nanotechnology-integrated electrochemical biosensors for the measurement of EVs and EV-related biomarkers.

**Figure 3:**
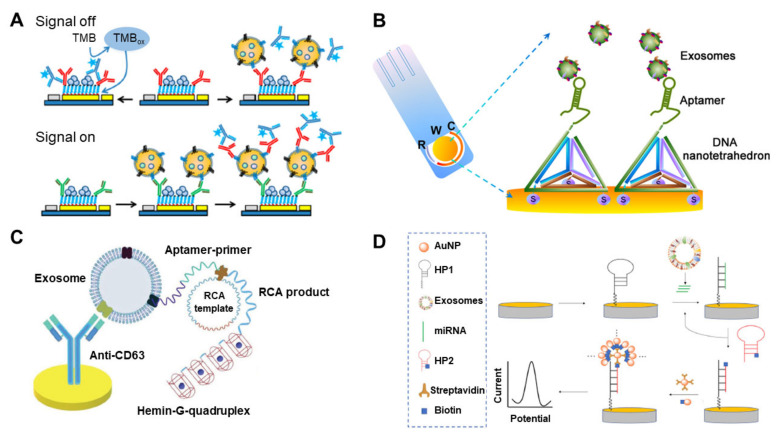
Electrochemical biosensor for EV detection. (**A**) Schematic diagram of the electrochemical sandwich immunosensor with an enzyme for measurement of EVs; (**B**) electrochemical aptamer-based sensor using DNA nanotetrahedron as a capture moiety on the electrode; (**C**) hemin/G-quadruplex-assisted signal amplification for sensitive electrochemical detection of EVs from gastric cancers; (**D**) signal amplification strategy for electrochemical EV detection by using hairpin DNA-functionalized silver nanoparticles. (reproduced with permission from [75], published by American Chemical Society 2016, reproduced with permission from [79] published by American Chemical Society 2017, reproduced with permission from [80] published by WILEY-VCH 2019 and reproduced with permission from [81] published by Elsevier 2020).

Among the several types of electrochemical biosensor for the detection of EVs, immunosensors, represented by ELISA, have been well-studied because of their inherent specific detection property. Doldan et al. exhibited the electrochemical sandwich immunosensor by using a surface marker-mediated signal amplification of down to four orders of magnitude compared with conventional ELISA (Figure 3A) [75]. Each EV has various surface markers, such as CD9 and CD63, detectable by the enzyme-tagged antibodies. The number of surface markers on the EV should proportionally exceed that of the EVs. In this context, the authors demonstrated the highly sensitive amperometric detection of EVs by measuring the electrochemically signal of HRP tagged to anti-CD9 [72]. The developed electrochemical biosensor measured EVs with a dynamic range of 2 × 10^2^–1 × 10^6^ particles/μL and could reliably quantify EVs in >1000-fold dilutions of human blood plasma. Cao et al. developed an amplified electrochemical biosensing system for human hepatoma (HepG2)-derived EV detection [76]. To amplify the electrochemical signal, the target EVs are concentrated on an anti-CD63-functionalized magnetic bead by magnetic separation and captured by a CD63 aptamer, which triggers a catalytic molecule machine that is dependent on a cascade toehold-mediated strand displacement reaction. The LOD of this system is 1.72 × 10^4^ particles/mL, with a linear range from 1 × 10^5^ to 5 × 10^7^ particles/mL. Moura et al. presented an electrochemical biosensor for EVs preconcentrated by immunomagnetic separation on anti-CDX-modified magnetic particles (CDX being CD24, CD44, CD54, CD326 or CD340), followed by labeling with the anti-CD63–HRP antibody as an electrochemical probe [77]. The EVs were derived from three breast cancer cell lines (MCF-7, SK-BR-3 and MDA-MB-231), and the specific receptors of the cancer-derived EVs were targeted by their respective antibodies. The developed electrochemical immunosensor reached a LOD of 10^5^ EVs/μL in human serum while avoiding the interference from free receptors in the serum matrix. An et al. developed a magneto-mediated electrochemical sensor based on host–guest recognition for the simultaneous analysis of breast cancer exosomal proteins [78]. CD63-aptamer-modified magnetic beads were used initially to capture the EVs. The expression levels of four proteins (mucin 1, human epidermal growth factor receptor 2, EpCAM and carcinoembryonic antigen) on the breast cancer patient-derived EVs were measured by using silica (Si) NPs modified with the respective aptamers and functionalized with a mercapto-ferrocene derivative. The sandwich structure (magnetic beads–EVs-Si NPs) was separated by a magnet and dissociated by adding agents that reduce disulfide bonds. A GO-cucurbit modified carbon electrode was then employed to measure the oxidation current signals, proportional to the quantity of EV. Results obtained from a clinical test of this approach confirmed higher levels of all four exosomal proteins in serum from a breast cancer patient compared with a healthy individual. The LOD was 1.2 × 10^3^/μL.

Besides the immunoaffinity-based methods, numerous studies have used an aptamer-based electrochemical sensor for EV detection. Wang et al. developed a nanotetrahedron-assisted aptasensor for capturing and detecting the surface proteins of hepatocellular EVs (Figure 3B) [79]. The oriented immobilized aptamers on the DNA nanotetrahedron improved the accessibility of the aptamer to EVs, providing an aptasensor with 100-fold higher sensitivity (10^4^/mL) when compared with the single-stranded aptamer-functionalized aptasensor (10^6^/mL). Dong et al. described a new strategy for EV detection based on aptamer recognition-induced multi-DNA release from magnetic particles and cyclic enzymatic amplification [82]. The multi-DNA release was triggered by the EV–aptamer interaction. Subsequent enzymatic degradation by Exo III exonuclease of functionalized DNA immobilized on the electrode turned the electrochemical signal off. The electrochemical signal reflected the concentration of the electro-active molecules—And this correlated with the multi-DNA concentration, which correlated with the EV concentration. As a result of the catalytic property of the Exo III exonuclease, a low LOD of 70 particles/μL was achieved, thereby demonstrating ultrasensitive detection with high selectivity. Huang et al. exhibited a label-free electrochemical aptasensor for the specific detection of gastric cancer EVs (Figure 3C) [80]. An anti-CD63-functionalized Au electrode captured the gastric cancer-derived EVs, and a specific gastric cancer EV aptamer with a G-quadruplex circular template triggered rolling circle amplification, producing multiple G-quadruplex units with HRP mimicking DNAzyme property. The aptasensor showed high selectivity and sensitivity of gastric cancer EVs and a LOD of 9.54 × 10^2^/mL. The electrochemical biosensor developed by An et al. was based on click chemistry of alkynyl-4-ONE and the DNA hybridization chain reaction (HCR) to amplify the signal by using a streptavidin–biotin reaction for the determination of tumor EVs [83]. The EV concentration was quantified in the range of 1.12 × 10^2^–1.12 × 10^8^ particles/μL by monitoring the electrochemical reduction current of 2,3-diaminophenazine generated from the oxidation of o-phenylenediamine by hydrogen peroxide. Zhao et al. developed an ultrasensitive electrochemical biosensor for the detection of EVs by coupling a 3D DNA walking machine, consisting of two specific DNA aptamers, with Exo III-assisted recycling [84]. Breast cancer cells (MCF-7) were detected with high sensitivity (1.3 × 10^4^ particles/mL) and specificity. In comparison, Yin et al. reported a slightly lower LOD of 1.2 × 10^4^ particles/mL by using an aptamer recognition-trigged label-free electrochemical biosensing method with Exo III-assisted signal amplification for highly selective and ultrasensitive detection of MCF-7-derived EVs [85]. The authors claimed that it could be shown the outstanding properties of simplicity, rapidness, cost-efficiency and easy manipulation.

Small non-coding RNAs, such as miRNA, are altered in cancers, suggesting that miRNAs could be reliable cancer biomarkers [86,87,88]. Therefore, electrochemical sensors have been also developed for the measurement of exosomal miRNAs. Boriachek et al. selectively isolated exosomal miRNAs from complex biological samples using magnetic beads pre-hybridized with biotinylated complementary probes and then directly capturing the exosomal miRNAs on the working electrode [89]. This amplification-free electrochemical biosensor displayed good reproducibility (% relative standard deviation [RSD] of <5.5), sensitivity (LOD of 1 pM) and dynamic range (1 pM–100 nM)) from various cancer cell lines and serum samples collected from patients with colorectal adenocarcinoma. Luo et al. exhibited a ratiometric electrochemical biosensor by using a locked nucleic acid (LNA)-modified electrode for the detection of exosomal miR-21, released by MCF-7 cancer cells [90]. The polylysine film-coated glassy carbon electrode was functionalized by a “Y”-shaped LNA, and the target exosomal miR-21 induced a conformational change in the “Y”-shaped LNA to the molecular beacon, which increases the redox peaks. This simple DNA structural transformation could detect exosomal miR-21 with a LOD as low as 2.3 fM. Guo et al. showed a sensitive electrochemical assay of miR-122 by HCR of the hairpin structure of the capture DNA on the surface of the electrode [91]. The miR-122 opened the hairpin DNA and triggered the HCR through the cross-opening and hybridization of two helper DNA hairpins. HCR induced the formation of long nicked double helixes, which captured the electro-active molecule and increased the electrochemical signal. The LOD for miR-122 detection was greatly improved to 53 aM offered high reproducibility. Cheng et al. developed an enzyme-free electrochemical biosensor for the detection of exosomal miRNAs by the double signal amplification strategy (Figure 3D) [81]. Ultrasensitive detection was achieved through a cyclic signal amplification method with the target miRNA (miR-21) and subsequent immobilization of silver (Ag) NPs on the electrode by a streptavidin–biotin interaction. A low LOD of 0.4 fM miR-21 in human biologic samples was demonstrated. Recent studies of electrochemical-based analytical methods for EV detection are compared in Table 3.

## 5. Raman-Based Analysis Methods for EV Detection

Raman spectroscopy is an optical analysis that measures the intensity of the inelastic scattering of the incident light to gather information about the chemical structures in individual molecules [92,93]. Raman spectra can be shifted along with the chemical structure of each molecule and specific Raman shifts can be used as a fingerprint of each molecule. Specific signals in Raman spectra can also be utilized as specific redox peaks in electrochemical biosensors, for determining the concentration of the target. Nonetheless, the Raman shift of typical biomarkers shows weak signal intensity, which necessitates biomedical applications, such as point-of-care testing or early diagnosis. Alternatively, surface-enhanced Raman scattering (SERS), which enhances the Raman signal intensity by a magnitude of 10^14^ because of the surface plasmon effects on the surface of metal nanomaterials, could overcome the limitation of low-intensity of Raman signals [94,95,96]. This section briefly introduces recent studies related to SERS-based exosomal detection platforms.

**Figure 4:**
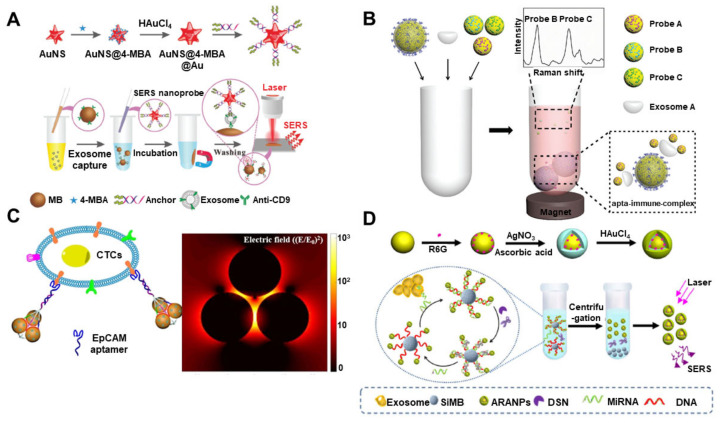
Raman-based biosensor for EV detection. (**A**) Sequential surface-enhanced Raman scattering (SERS)-based assay process for the detection of EVs by using SERS nanoprobes (AuNS@4-MBA@Auanchor); (**B**) multiple detections of cancer EVs through aptamer-functionalized Au nanoparticle and magnetic bead; (**C**) aptamer-functionalized Au nanoparticles in a triangular pyramid for the signal amplification of SERS biosensor for EV detection (**D**) duplex-specific nuclease (DSN)-assisted SERS signal amplification for quantitative detection of exosomal miRNA using Au@Ag–Au alloy shell nanoparticles. (Reproduced with permission from [97], published by the royal society of chemistry 2018, reproduced with permission from [101] published by the royal society of chemistry 2018, reproduced with permission from [102] published by American Chemical Society 2019 and reproduced with permission from [103] published by Elsevier 2018).

For the capture and detection of EVs using Raman-based biosensors, the antibody-mediated immunoassay is one of the representative methods. Tian et al. exhibited a highly sensitive scattering SERS-based EV biosensor by combining SERS nanoprobes and portable Raman devices (Figure 4A) [97]. In this immunosensor system, Au nanostar@4-mercaptobenzoic acid@nanoshell structures functionalized with a bivalent cholesterol-labeled DNA were utilized as a Raman probe. The targeted EVs were immunomagnetically captured by anti-CD9-functionalized magnetic beads. Afterward, the SERS nanoprobes (Au nanostar@4-mercaptobenzoic acid@nanoshell) were anchored to the EV membrane through hydrophobic interactions. The LOD of this SERS-biosensor was 27 particles/μL. The authors claimed that the developed biosensor was easily fabricated and had considerable potential for early diagnosis, among various other applications. Kwizera et al. exhibited a miniaturized affinity-based SERS-sensitive device to capture and analyze EVs in a target-specific manner with the assistance of low-cost 3D printing technology [98]. QSY21-coated Au nanorods were used as Raman reporters to quantitatively detect the surface marker on the EVs derived from HER2-positive breast cancer. This analytical platform provides precise detection with a LOD of 2 × 10^6^ EVs/mL and evaluates over 80 samples within 2 h, simultaneously. Zhang et al. developed a SERS-based EV profiling platform for simultaneous measurement and detection of multiple EV membrane proteins, including glypican-1 [99], EpCAM and CD44 variant isoform 6 (CD44V6), isolated from pancreatic carcinoma cells. For the capture and probe of specific EVs, diverse antibody-functionalized Au NPs were applied to the medium containing the EVs. This highly sensitive SERS platform measured as low as 2.3 × 10^6^ particles/mL in a small sample (10 μL) and showed high specificity profiling of the three biomarkers. In addition, phenotypic profiling of EVs from colorectal cancer, pancreatic cancer and bladder cancer cell lines (SW480 and C3) was conducted that successfully classified the EVs in both phosphate-buffered saline and plasma. Pang et al. developed a SERS immunoassay for the capture and analysis of exosomal programmed death ligand 1 (PD-L1), which is a predictor for antiprogrammed death-1 therapy [100]. Anti-PD-L1-functionalized Fe_3_O_4_@TiO_2_ NPs were used to capture and separate the specific EVs by magnetic force and the Au@Ag@4-mercaptobenzoic acid nanoprobe for SERS analysis was bound to the EV as a sandwich immunoassay. Exosomal PD-L1 could be measured with a LOD of 1 PD-L1 EV/μL in 4 μL of undiluted serum within 40 min.

Aptamer-based EV detections by SERS analysis were also developed recently. Wang et al. demonstrated a SERS-based method for the detection of multiple EVs by using a magnetic bead-coated Si shell and Au (methylene blue@SiO_2_@Au) decorated with the aptamer of a generic surface protein, CD63 (Figure 4B) [101]. The LOD was 32, 73 and 203 EVs/μL for breast cancer (SK-BR-3), colorectal cancer (T84) and prostate cancer (LNCaP) EVs, respectively. Based on the results, the proposed SERS-based biosensor with aptamer is promising to diagnose early stage cancers. Zhang et al. developed a novel Raman probe composed of Au NPs and triangular pyramid DNA for the sensitive detection of circulating tumor cells and EVs (Figure 4C) [102]. Triangular pyramid DNA could induce electromagnetic hot spots, where an intense electromagnetic effect through the triangular arrangement of three AuNPs is. As a result, 5−100,000 cells/mL of HeLa cells could be detected in 1.0 × 10^6^ cells/mL HEK-293T cells without the enrichment process. For the EV, the LOD was 1.1 × 10^2^ particles/μL with high specificity, and the assay performed well in plasma samples. Zhu et al. described a sensitive aptasensor by using a hydrophobic-assembled nanoacorn (HANA) with Au@Ag nanocubes as a Raman probe [104]. Exosomal proteins, including CD63, HER2 and EpCAM, could bind to the aptamers on the surface of HANA, and the Au@Ag nanocube attached to the vacant HANA through electrostatic interactions. This well-oriented distribution of SERS probes confirms outstanding repeatability, whereas the accurate sub-nanometer junctions assure high sensitivity. Measurement of HER2 was highly sensitive and reproducible (RSD < 7%), and EVs were detected at 50 EVs/μL with a low RSD of 6.4% [104]. Ning et al. developed a reliable SERS sensor for the simultaneous detection of multiple cancer-related EVs [105]. The SERS probes consisted of Au–Ag–Ag core–shell–shell nanotrepangs (GSSNTs) and functionalized DNA with a bumpy structured surface. With three different Raman probe-modified GSSNTs, multiple EVs from different cancer cell lines (LNCaP, SK-BR-3 and HepG2) were successfully measured with high sensitivity and excellent multiplexed detection capacity. The quantitative detection of every single EV showed a linear correlation ranging from 1 to 10^4^ particles/μL.

Exosomal miRNAs can also be measured by the Raman-based analytical methods with suitable nanomaterials for signal enhancement. Ma et al. exhibited a SERS-based exosomal miRNA sensor with duplex-specific nuclease-assisted signal amplification and Au@R6G@AgAu NPs, where R6G is rhodamine 6G, as a strong Raman reporter (Figure 4D) [103]. Au@R6G@AgAu NPs were connected to Si microbeads through the DNA capture probe, which could be complimentarily bound to the target miRNA. If the target miRNA binds to the DNA capture probe, Au@R6G@AgAu is dissociated from the Si microbead by the duplex-specific nuclease, and the SERS signal is increased proportionally to the exosomal miRNA level. The LOD was just 5 fM, and the method could differentiate the patients from healthy individuals, showing a more than six-fold difference in the signal. Lee et al. developed a uniform plasmonic head-flocked Au nanopillar substrate for the signal enhancement of SERS by providing multiple hotspots [106]. Two different LNAs captured and detected the target miRNA with Cy3 labeling. Using this Au nanopillar array, an extremely low detection of three different miRNAs (miR-21, -222 and 200c) was achieved with a wide dynamic range (1 aM–100 nM). In addition, a reliable expression pattern of exosomal miRNA from breast cancer cell lines was obtained, and different breast cancer subtypes could be differentiated. Recent studies of Raman-based analytical methods for EV detection are compared in Table 4.

## 6. Conclusions and Future Perspective

This review article focused on biosensors that integrate nanotechnology with diverse analytical methods, including SPR-, fluorescence-, electrochemical- and Raman-based strategies for the effective measurement of EVs as biomarkers in diagnosing several diseases. Due to the inherent properties of nanomaterials (high surface-to-volume ratio, high reactivity and signal-enhancing effect), the precise and sensitive detection of EVs could be demonstrated. In addition, specific properties and multifunctionalities of each nanomaterial could facilitate the efficient measurement of EVs present in real samples. Most of the presented EV biosensors showed improved sensitivity by using functionalized nanomaterials and unique strategies. Besides the four representative analytical methods, others, such as field-effect-transistor [107,108], mechanical changes using a cantilever or quartz crystal microbalance [109,110,111] and giant magnetoresistance [112] could also be integrated with nanotechnologies to measure EVs. In this context, nanotechnology-integrated detection systems could be expanded to diverse analytical methods. In addition, label-free detection of EV can be developed by exploiting the high sensitivity of nanomaterials. One study presented a nanogap-integrated plasmonic sensing platform for EV detection without a Raman probe labeling step [113]. For improving the sensitivity, they used nanogap combining sub-volt dielectrophoretic trapping and Au NPs for real-time SERS imaging. Another study fabricated graphene-functionalized Au nanopyramids on SiO_2_ and successfully characterized EVs isolated from different biologic sources using unbiased principal component analysis [114].

Besides detecting EVs by their specific surface proteins, encapsulated biomolecules, such as miRNA, mRNA and protein, could be important biomarkers for the diagnosis of diseases. Some exosomal miRNAs have been utilized as cancer biomarkers [115,116,117]. However, the expression levels of one miRNA could not indicate the presence of diseases precisely. Therefore, profiling of exosomal miRNA or other biomolecules will be more important for obtaining precise and personalized information, as well as diagnostic information or drug sensitivity. To this end, the isolation, rupture, and other pretreatment steps should be integrated into the sensing platform for collecting the encapsulated biomolecules efficiently. A powerful tool to leverage the functionality of biosensors is artificial intelligence-driven multi-technology bioprinting systems. On this wise, isolation, rupture, and other pretreatment steps should be integrated on the sensing platform for collecting the encapsulated biomolecules efficiently, as well as detection of the EV itself precisely. EV presents in diverse body fluids, such as blood, saliva, urine and interstitial fluids. For efficient measurement of EV in several real samples, preparation steps are very important to prevent pulse-positive signals and reduce trivial noise. Therefore, integrated platforms of sample preparation, detection, and analysis modules will be further developed with various nanotechnology to improve their performances. The concept of “organ-on-a-chip” is emerging for the etiology and drug screening of several diseases. For efficient drug testing, EV biosensors as a sensing module can provide real-time monitoring and fast response to the drugs on disease-emulated “organ-on-a-chip.” Currently, “organ-on-a-chip” operates as a proxy for in vitro and in silico research.

In conclusion, nanotechnology can be combined with EV biosensors to improve sensing capabilities, such as high selectivity, high sensitivity, straightforward and rapid detection, multi-detection and in situ monitoring. Here, we report the present status in the integration of nanotechnology-based biosensor platforms and analytical methods (SPR-, fluorescence-, electrochemical-based and Raman-based measurements) for detecting EVs. Each method has unique properties and suitable nanotechnologies are effortlessly utilized to improve the sensing performances. On this wise, isolation, rupture, and other pretreatment steps should be integrated on the sensing platform for collecting the encapsulated biomolecules efficiently, as well as detection of the EV itself precisely. EV presents in diverse body fluids, such as blood, saliva, urine and interstitial fluids. For efficient measurement of EV in several real samples, preparation steps are very important to prevent pulse-positive signals and reduce trivial noise. Therefore, integrated platforms of sample preparation, detection, and analysis modules will be further developed with various nanotechnology to improve their performances. In the immediate future, it is expected that micro total analysis systems and advanced “organ-on-a-chip” platforms with nanomaterial-based sensing modules for EVs will provide a robust in vitro drug development in platforms that can replace in vitro cell culture models and in vivo animal models and enable in vitro personalized investigations. Recently, multifunctional nanomaterials have been exploited to possess remarkable properties, such as enhancement of electron transfer reactions and improvement of quantum yield, for sensitive detection of optical properties. The development of nanotechnology will offer innovative and creative directions to develop novel biosensing platforms for EVs to increase the full recovery rate of diseases through the early diagnosis at a low level of exosomal biomarkers in real samples.

## Figures and Tables

**Table 1:** Comparison of Surface Plasmon Resonance (SPR)-Based Bionano Sensors for EV Detection.

Method	Working Principle	Target	Correlation Range	Detection Limit	Ref
SPR	TiN film functionalized by biotinylated anti-epidermal growth factor receptor variant-III (EGFRvIII) antibodies	Glioma cells (U251)	0.005–500 µg/mL	2.75 × 10^−3^ µg/mL	[41]
	Dual signal amplification via two-step hybridization using aptamer functionalized Au NPs	MCF-7 breast cancer cells and MCF-10A normal breast cells	Not stated	50 EVs/μL	[42]
LSPR	Periodic nanohole arrays with gold layer functionalized antibodies	Ovarian cancer	4.03 × 10^2^–1.32 × 10^6^ EVs μL	4.03 × 10^2^ EVs/μL	[45]
	Self-assembled Au NIs without functionalization	A-549 and SH-SY5Y cells	0.194–100 μg/mL	0.194 μg/mL	[43]
	Au nanoplasmonic array functionalized with anti-CD63 antibody	Breast cancer	Not stated	1 × 10^2^ EVs/μL	[46]

**Table 2:** Comparison of Colorimetric/Fluorescence-Based Bionano Sensors for EV Detection.

Method	Working Principle	Target	Correlation Range	Detection Limit	Ref
Colorimetric	ZnO-nanowires-coated three-dimensional (3D) scaffold chip	Breast cancer (MCF-7)	2.2 × 10^5^–2.4 × 10^7^ EVs/μL	2.2 × 10^4^ EVs/μL	[52]
	microfluidic chip with self-assembled three-dimensional herringbone nanopatterns (nano–HB)	Ovarian cancer	1 × 10^3^–5 × 10^5^ EVs/μL	10 EVs/μL	[53]
	graphitic carbon nitride nanosheets (g-C3N4 NSs)	Breast cancer (MCF-7)	0.19 × 10^7^–3.38 × 10^7^ EVs/μL	1.352 × 10^3^ EVs/μL	[54]
	aptamer-CD63 functionalized single-walled carbon nanotubes (s-SWCNTs)	Breast cancer (MCF-7)	1.84 × 10^6^–2.21 × 10^7^ EVs/μL	5.2 × 10^2^ EVs/μL	[56]
	EpCAM aptamer modified Fe_3_O_4_ NPs	Prostate cancer	0.4 × 10^5^–6.0 × 10^5^ EVs/μL	3.58 × 10^3^ EVs/μL	[57]
	Au NPs complexed with a panel of aptamers	Prostate cancer	0–12.8 μg/mL	Not stated	[58]
	PLA–RPA–TMA assay	Nasopharyngeal carcinoma cell	0.1–10^5^ EVs/μL	0.1 EVs/μL	[59]
Fluorescence	Fluorescent labeled aptamer/GO nanoprobe	Prostate cancer	1.6 × 10^2^–1.6 × 10^5^ EVs/μL	1.6 x 10^2^ EVs/μL	[65]
	Cy3 labeled aptamer–CD63 and Ti_3_C_2_ MXenes complex	Melanoma (B16), Breast cancer (MCF-7), ovarian carcinoma (OVCAR-3), liver cancer (Hep G2)	10–10^6^ EVs/μL	0.1 EVs/μL	[61]
	Aptamer modified UCNP and Au NRs	liver cancer (Hep G2)	1.0 × 10^4^–1.0 × 10^9^ EVs/μL	1.1 × 10^3^ EVs/μL	[62]
	Multifunctional magneto-plasmonic NRs	Neurogenesis(miR-124)	1 Pm–10^6^ pM	1 pM	[63]
	Copper-mediated fluorescent signal amplification	Cancer (Hep G2)	7.5 × 10^4^ to 1.5 × 10^7^ EVs/μL	4.8 × 10^4^ EVs/μL	[64]

**Table 3:** Comparison of Electrochemical Bionano Sensors for EV Detection.

Method	Working Principle	Target	Correlation Range	Detection Limit	Ref
Electrochemical	Electrochemical sandwich immunosensor (amperometry)	Breast cancer (MCF-7)	2 × 10^2^–1 × 10^6^ EVs/μL	2 × 10^2^/μL	[75]
	Cascade toehold-mediated strand displacement reaction (CTSDR) (voltammetry)	Liver cancer (HepG2)	1 × 10^3^ to 5 × 10^5^ EVs/μL	1.72 × 10^2^/μL	[76]
	Electrochemical immunosensor using magnetic bead (amperometry)	Breast cancer (MCF7, MDA-MB-231 and SK-BR-3)	1 × 10^2^–1 × 10^6^ EVs/μL	10^2^/μL	[77]
	Electrochemical sensor based on graphene oxide-cucurbit modified carbon electrode (voltammetry)	Breast cancer (MCF-7, SK-BR-3, MDA-MB-231 and BT474)	1.2 × 10^3^–1.2 × 10^7^ EVs/μL	1.2 × 10^3^/μL	[78]
	Electrochemical aptasensor using DNA nanotetrahedron and aptamer (voltammetry)	Liver cancer (HepG2)	1 × 10^2^–1 × 10^9^ EVs/μL	10^2^/μL	[79]
	Electrochemical aptasensor by cyclic enzymatic amplification (voltammetry)	Prostate cancer and breast cancer (LNCaP and MCF-7)	70 to 1 × 10^5^ EVs/μL	70/μL	[82]
	G-quadruplex circular template triggered rolling circle amplification (RCA) for electrochemical sensor (voltammetry)	Gastric cancer (GES-1 and SGC7901)	0.954–7.8 × 10^3^ EVs/μL	0.954 /μL	[80]
	Electrochemical biosensor based on click chemistry of alkynyl-4-ONE (voltammetry)	Breast cancer (MCF-7)	1.12 × 10^2^–1.12 × 10^8^ EVs/μL	1.12 × 10^2^/μL	[83]
	Electrochemical biosensor by coupling the DNA walking machine (voltammetry)	Breast cancer (MCF-7)	13–1.0 × 10^7^/μL	13/μL	[84]
	Exo III-assisted cycling reaction for signal amplification (voltammetry)	Breast cancer (MCF-7)	12–3.4 × 10^5^ EVs/μL	12/μL	[85]
	Electrochemical detection of exosomal miRNAs by using magnetic separation (voltammetry)	miR-21	1 pM–100 nM	1 pM	[89]
	Electrochemical biosensor by Y-shaped locked nucleic acid (LNA) (voltammetry)	miR-21	10–70 fM	2.3 fM	[90]
	Electrochemical assay of miR-122 by hybridization chain reaction (HCR) (voltammetry)	miR-122	1 × 10^2^–1 × 10^11^ aM	53 aM	[91]
	Enzyme-free electrochemical biosensor by the double signal amplification strategy (voltammetry)	miR-21	1 fM–200 pM	0.4 fM	[81]

**Table 4:** Comparison of Raman-Based Bionano Sensors for EV Detection.

Method	Working Principle	Target	Correlation Range	Detection Limit	Ref
Raman	SERS-based immunosensor by gold nanostar@4-mercaptobenzoic acid@nanoshell structures	Liver cancer (HepG2)	40–4 × 10^7^ EVs/μL	27/μL	[97]
	Miniaturized affinity-based SERS-sensitive device by 3D printing	Breast cancer (MDA-MB-231, MDA-MB-468 and SK-BR-3)	10^6^–10^8^ EVs/μL	2×10^3^/μL	[98]
	SERS-based EV profiling platform using surface proteins	Colorectal cancer and bladder cancer (SW480 and C3)	2.3 × 10^3^–2.3 × 10^8^ EVs/μL	2.3×10^3^/μL	[99]
	SERS immunoassay using anti-PD-L1-functionalized Fe_3_O_4_@TiO_2_ nanoparticles	Lung cancer (A549)	5–2 × 10^2^ EVs/μL	1/μL	[100]
	SERS-based multi-EV detection by MB@SiO_2_@Au nanoparticle	Breast cancer and prostate cancer (SK-BR-3, T84 and LNCaP)	32–3.2 × 10^5^ EVs/μL	32/μL	[101]
	SERS-based aptasensor by Au nanoparticles and triangular pyramid DNA	Breast cancer and cervical cancer (MCF-7, Hela and HEK-293 T)	1.0 × 10^3^–1.0 × 10^7^ EVs/μL	1.0 × 10^3^/μL	[102]
	SERS biosensor with hydrophobic assembled nanoacorn and Au@Ag nanocubes	Breast cancer (MCF7, MBA-MD-231)	50–1.0 × 10^6^ EVs/μL	50/μL	[104]
	SERS aptasensor by gold–silver–silver core–shell–shell nanotrepangs	Breast cancer, prostate cancer and liver cancer (SK-BR-3, HepG2 and LNCaP)	1–10^4^ EVs/μL	1/μL	[105]
	SERS-based sensor with duplex-specific nuclease (DSN) and Au@R6G@AgAu nanoparticles	miR-21	5 fM–20 pM	5 fM	[103]
	Uniform plasmonic head-flocked gold nanopillar substrate for the signal enhancement of SERS	miR-21, 222, 200c	1 aM–100 nM	1 aM	[106]
